# Landiolol reduces hemodynamic responses to bronchoscopy-assisted suctioning in intubated ICU patients

**DOI:** 10.1186/2052-0492-2-6

**Published:** 2014-01-23

**Authors:** Junpei Tochikubo, Yushi U Adachi, Tadashi Ejima, Atsushi Numaguchi, Naoyuki Matsuda, Shigehito Sato, Norihiko Shiiya

**Affiliations:** First Department of Surgery, Hamamatsu University School of Medicine, 1-20-1 Handayama, Higashi-ku, Hamamatsu, 431-3192 Japan; Department of Emergency and Critical Care Medicine, Nagoya University Graduate School of Medicine, 65 Tsurumai-cho, Showa-ku, Nagoya, 466-8550 Japan; Department of Emergency Medicine, Nagoya University Hospital, 65 Tsurumai-cho, Showa-ku, Nagoya, 466-8550 Japan; Department of Pediatrics and Developmental Pediatrics, Nagoya University Graduate School of Medicine, 65 Tsurumai-cho, Showa-ku, Nagoya, 466-8550 Japan; Department of Anesthesiology and Resuscitation, Hamamatsu University School of Medicine, 1-20-1 Handayama, Nagoya, 431-3192 Japan

**Keywords:** Landiolol, Fiber bronchoscopy, Hemodynamic response, Endotracheal suctioning

## Abstract

Landiolol is an ultra-short-acting β_1_-selective antagonist developed in Japan that was recently approved for the treatment of tachycardia in intensive care units (ICUs). This study investigated the protective effects of landiolol against the cardiovascular responses during bronchoscopic endotracheal suctioning. This study enrolled 15 patients requiring orotracheal intubation in an ICU. All of the patients required endotracheal suctioning using fiber bronchoscopy while sedated at a Ramsay Scale of 2–3. All subsequent suctioning procedures were assigned randomly to three groups using a cross-over design: saline as a placebo (group C) or 20 or 40 μg kg^-1^ min^-1^ landiolol, respectively (groups L20 and L40). The infusion was started 3 min before bronchoscopy and continued for 6 min. The central venous pressure (CVP) heart rate (HR) and arterial blood pressure (BP) were recorded. Fourteen patients completed the investigation, and 30 procedures (*n* = 10/group) were analyzed. The suctioning significantly increased the CVP, HR, and BP in groups C and L20, although the changes in BP were of shorter duration in group L20. No significant increase in the hemodynamic parameters was observed in group L40. The administration of landiolol 40 μg kg^-1^ min^-1^ prevented a harmful hyperdynamic circulatory response to bronchoscopic endotracheal suctioning, without obvious decreases in HR or BP after the intervention.

## Findings

In patients with orotracheal intubation and mechanical ventilation at intensive care unit, the supplemental administration of landiolol effectively diminished harmful hemodynamic changes during bronchoscopic endotracheal suctioning.

## Introduction

Landiolol is an ultra-short-acting, β_1_-selective adrenergic antagonist developed in Japan [1, 2]. The reported half-life is approximately 4 min. Recently, landiolol became available for controlling the heart rate in intensive care units (ICUs) in Japan [3]. The results of a large, prospective, multicenter, randomized trial of controlling rapid heart rate were published as the J-Land Study [4].

Patients who have undergone tracheal intubation and mechanical ventilation often require endotracheal suctioning to remove sputum and secretions from the lungs, and this stimulus induces unpleasant cardiovascular sympathetic responses [5]. A rising heart rate increases the risk of myocardial ischemia [6]. This study investigated the effects of short-term pre-administration of landiolol on the hemodynamic responses during endotracheal bronchoscopic suctioning in ICU patients.

## Methods

A prospective double-blind control study using a cross-over design was conducted after obtaining approval from the Institutional Review Board of Hamamatsu University School of Medicine (registration number 18–164, Ethics Committee of Medicine). All interventional procedures, including written informed consent from the participants, conformed to accepted study protocols.

The study enrolled 15 patients requiring orotracheal intubation for more than 96 h in the ICU. All patients were sedated to maintain their comfort using fentanyl, propofol, and dexmedetomidine, and intensivists usually tried to interrupt the sedatives each morning and restarted the administration of sedatives in the evening. The participants required endotracheal suctioning using a fiber bronchoscope while sedated at a Ramsay sedative score of 2–3 from the evening to the next morning. The drugs being administered were unchanged for 1 h before the bronchoscopic suctioning and maintained until the termination of the intervention. During the study period, the subsequent suctioning procedures were assigned to three regimens: saline as a placebo (group C) or 20 or 40 μg kg^-1^ min^-1^ landiolol (groups L20 and L40). The infusion was started 3 min before bronchoscopy and continued for 6 min. The heart rate (HR), invasive arterial blood pressure (BP), and central venous pressure (CVP) were recorded.

The patients' demographics and changes in cardiovascular parameters were compared among the groups using analysis of variance. A probability of less than 0.05 was considered statistically significant, and the differences were subsequently analyzed using the Newman-Keuls *post hoc* multiple comparisons test.

## Results

Fourteen patients completed the investigation, and 30 procedures (*n* = 10/group) were analyzed. Only two patients received three regimens, and the others experienced two. The patients' demographic data and a study flow diagram are shown in Table 1. Throughout the study period, no adverse unexpected complication was observed.

**Table 1:** **Demographic data of the patients**

Demographic data	Number
Age	73 ± 6
Sex (m/f)	9/5
Weight (kg)	53 ± 10
Height (cm)	160 ± 12
Heart rate (bpm)	85 ± 12
Systolic blood pressure (mmHg)	122 ± 13
Diastolic blood pressure (mmHg)	60 ± 11
Central venous pressure (mmHg)	7.0 ± 4.8

**Figure 1:**
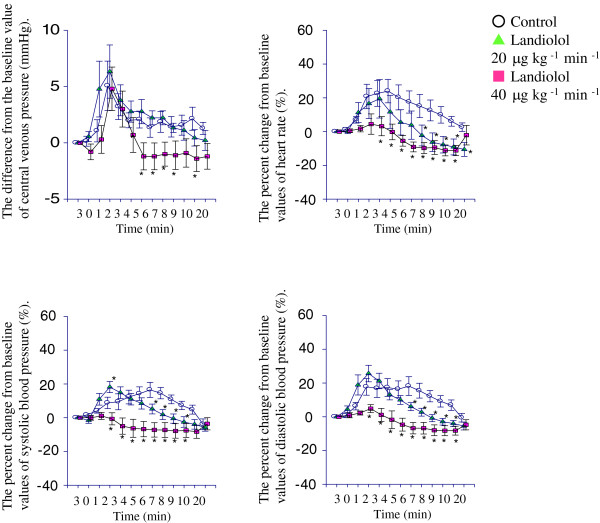
**Results of changes in central venous pressure (CVP), heart rate (HR), and systolic and diastolic pressures.** The change in CVP was expressed as differences from the baseline values (mean ± SE). Other results were expressed as means (SE) of percent change from the baseline values. *p < 0.05 vs*.* control group.

Bronchoscopic suctioning significantly increased the CVP immediately after the event in all groups, and this increase lasted for up to 3 min after the stimulus (Figure 1). In groups C and L20, the HR and BP increased significantly immediately after suctioning. A few minutes after suctioning, the values of the cardiovascular parameters were lower in group L20 than in group C. In group L40, the HR and BP were significantly lower than those in group C throughout the period. There were no significant differences in the measured parameters among the three groups 20 min after suctioning.

## Discussion

Supplemental administration of landiolol reduced the cardiovascular responses to noxious stimuli of bronchoscopic suctioning. The larger dose of landiolol was more effective than the smaller dose. The increase in CVP immediately after suctioning was identical in all three groups; therefore, the effect of the noxious stimulus on the patients was considered comparable. Increasing intrathoracic or intra-abdominal pressure induced by physiological responses was observed in all patients.

The main effect of a β_1_-selective adrenergic antagonist on hemodynamic parameters is a decrease in heart rate. Nevertheless, this investigation showed that landiolol effectively reduced the increasing BP following a noxious stimulus. The larger dose (40 μg kg^-1^ min^-1^) completely abolished the hypertensive reaction. The major advantage of a β-blocker for controlled hypotension is the lack of both responsive tachycardia to the hypotension and responsive hypertension against bradycardia.

In this study, the administration of landiolol was effective in preventing tachycardia and hypertension without unfavorable circulatory depression after the termination of bronchoscopic suctioning. Even with the larger dose, all the measured parameters recovered within 20 min after the infusion. The ICU patients sometimes receive continuous infusions of vasopressors to maintain a normal HR and BP. Upon a sudden decrease in HR and BP, intensivists usually increase the rate of catecholamine infusion. However, frequent changes in the infusion require more intensive monitoring and human resources. Therefore, prolonged circulatory depression resulting from prophylaxis to the response to invasive stimuli might have more detrimental consequences.

The limitations of this study should be addressed. First, the participants were already receiving sedatives and analgesics, so we could not evaluate the precise effect of landiolol itself. Second, the results for each patient were consistent and comparable; however, the sample size was small. The adverse effect of landiolol should be appraised. Therefore, further studies are required. The study showed clinical advantages of landiolol for controlling hemodynamic changes against noxious stimuli.

## Authors’ information

JT is an intensivist and surgeon of Hamamatsu University School of Medicine. YUA worked in the Intensive Care Unit of Hamamatsu University School of Medicine, from August 2005 to January 2010. SS is the concurrent Director of the Intensive Care Unit of Hamamatsu University School of Medicine. TE and AN are intensivist and ER physicians at the Nagoya University Hospital. NM is the chairman of the Nagoya University Graduate School of Medicine. NS is a chairman and director of the Hamamatsu University School of Medicine.
